# Implications of Assisted Human Reproduction During Coronavirus Disease 2019 (COVID-19) Pandemic

**Published:** 2020

**Authors:** Mohammad Reza Sadeghi

More than six months have been passed since the outbreak of coronavirus disease (Covid-19) and during this short period, the virus quickly and extensively has been spread worldwide. The human virus has been known since 1960s. But over the past half-century, little attention has been paid to the virus. At recent, more than 40 coronaviruses have been identified in this family that most of them infected animals. Few of them are causes of human diseases such as common cold (15% of cases), severe acute respiratory syndrome (SARS-CoV), Middle East respiratory syndrome coronavirus (MERS-CoV) and severe acute respiratory syndrome coronavirus 2 (SARS-CoV-2). In spite of living with most of coronavirus family and their circulation among the human population for a very long time, we know very little about most members of this family especially the new corona-virus, Covid-19. Every day, there are reports of the Covid-19 disease with varying degrees of severity, from flu-like symptoms to death that have not been stated previously with other coronaviruses (1).

The known coronavirus only leads to acute respiratory syndrome in human, but the new coronavirus is attached to ACE2 as its specific receptor. In addition to respiratory system, ACE2 is present in other tissues including kidney, gastrointestinal tract, and blood vessels (Arteries and veins). Therefore, pathogenesis of Covid-19 has occurred in organs other than the lungs. Due to the adverse effects and unknown behavior of coronavirus, from the beginning of Covid-19 outbreak, it was recommended to suspend the unnecessary and elective medical interventions, such as assisted reproductive technologies. ACE2 receptor has not been found on sperm and oocyte. Moreover, vertical transmission and severe prenatal complications of coronavirus on pregnant women have never been reported. However, the rate of preterm labor slightly increased which may be attributed to altering of immune privilege between mother-fetus and disturbance of normal pregnancy. Consequently, occurrence of natural pregnancy has not been prohibited during Covid-19 outbreak (2).

In this issue, you will find a systematic review entitled “Coronavirus Disease 2019 (COVID-19): A Systematic Review of Pregnancy and the Possibility of Vertical Transmission” that represents all studies on this subject until now.

However, due to the lack of knowledge about symptoms and pathogenesis of SARS-CoV-2 on the fetus, infants and children, infertility treatment was almost completely stopped following outbreaks. Some medical interventions to treat infertility require the transfer of gametes and embryos. Yet, there is the possibility of virus transmission to a healthy mother through in vitro fertilization wherein gametes and embryos journey a long way out of body. Although there is no evidence of couple’s infection with coronavirus, the application of such technologies is intermitted temporarily to discover more about the behavior of the virus (3).

In addition, another concern is the direct effects of the virus on the reproductive system due to the unknown pathogenesis of Covid-19. Preliminary studies have showed the presence of ACE2 on reproductive cells and tissues which leads to impairment of female and male fertility. High levels of ACE2 is expressed in testis on spermatogonia (Spg), leydig cells (LC) and Sertoli cells (SC) as the potential route for coronavirus entry into these cells. However, several studies have not found SARS-CoV-2 in semen following acute infection, but elevated LH level as a sign of primary hypogonadism is reported. Future studies may show orchitis like consequences of SARS-CoV-2 infection on testis and spermatogenic cells. We may find similar consequences and pathogenesis in the female reproductive system in the future (4).

Although infertility does not threaten the survival of infertile couples, it may have severe impact on their quality of life in future. Human fertility, especially in women, can be affected by a number of age-dependent factors. Most of pathophysiological effects on fertility potential are irreversible; therefore, appropriate actions at proper time can prevent irreversible childlessness of infertile couples. Recently, infertility treatment and assisted reproduction services were resumed along with protection guidelines for clients and health care providers following the transition of Covid-19 outbreak from the log phase to the decline phase and control of the disease in most countries. Therefore, at present, we should prevent the spread of the disease from symptomatic and asymptomatic patients to clinic’s staff, employees; to top it all off, rigorous screening for couples seeking infertility treatment is highly recommended. Currently, the best strategy is making an interval between implementation of assisted reproductive technology (ART) and start of pregnancy. Accordingly, controlled ovarian stimulation, ovum pickup, fertilization and cryopreservation of embryos can be performed without any delay; however, embryo transfer will be delayed till subsiding of the pandemic as the best policy for infertility clinics.
